# Dynamics and stabilization of the rumen microbiome in yearling Tibetan sheep

**DOI:** 10.1038/s41598-019-56206-3

**Published:** 2019-12-23

**Authors:** Lei Wang, Ke Zhang, Chenguang Zhang, Yuzhe Feng, Xiaowei Zhang, Xiaolong Wang, Guofang Wu

**Affiliations:** 1 Academy of Animal Science and Veterinary Medicine, Qinghai University, Xining, 810016 China; 2 State Key Laboratory of Plateau Ecology and Agriculture, Qinghai University, Xining, 810016 China; 3 College of Animal Science and Technology, Northwest A&F University, Yangling, 712100 China

**Keywords:** Applied microbiology, Microbiome

## Abstract

The productivity of ruminants depends largely on rumen microbiota. However, there are few studies on the age-related succession of rumen microbial communities in grazing lambs. Here, we conducted 16 s rRNA gene sequencing for bacterial identification on rumen fluid samples from 27 Tibetan lambs at nine developmental stages (days (D) 0, 2, 7, 14, 28, 42, 56, 70, and 360, n = 3). We observed that *Bacteroidetes* and *Proteobacteria* populations were significantly changed during the growing lambs’ first year of life. *Bacteroidetes* abundance increased from 18.9% on D0 to 53.9% on D360. On the other hand, *Proteobacteria* abundance decreased significantly from 40.8% on D0 to 5.9% on D360. *Prevotella_1* established an absolute advantage in the rumen after 7 days of age. The co-occurrence network showed that the different microbial of the rumen presented a complex synergistic and cumbersome relationship. A phylogenetic tree was constructed, indicating that during the colonization process, may occur a phenomenon in which bacteria with close kinship are preferentially colonized. Overall, this study provides new insights into the colonization of bacterial communities in lambs that will benefit the development of management strategies to promote colonization of target communities to improve functional development.

## Introduction

Rumen microbes are essential for ruminant health. Ruminants rely upon the action of microbial fermentation in the rumen to digest plant fibers and convert some nutrients that cannot be directly utilized into animal proteins for host utilization^1^. There are many factors leading to changes in rumen microbial communities, primarily diet^2^, living environment, and host genotype^3^. Diurnal communities within the rumen microbiome exhibit oscillatory patterns following feeding and metabolites produced by the microbiome condition the rumen environment, leading to dramatic diurnal changes in community composition and function^4^. An earlier study investigated the genomic signatures of niche specialization in the rumen microbiome^5^. Another study explored structural and functional elucidation of the rumen microbiome influenced by various diets and microenvironments, providing deeper insights into the complicated network of bacterial interactions and adaptations to various substrates^6^. Although considerable efforts have focused on cataloguing the adult rumen microbiome and its relationship to complex diets^7,8^, studies on the lamb rumen microbiota have been restricted to 16 S rRNA amplicon sequencing, and/or different diets and hosts^9,10^. The processes shaping the rumen microbiota in early development have not been examined, particularly in groups of lambs that follow ewes for grazing.

The management of breeding sheep in high-altitude grazing areas is extensive and feeding practices are based on ecological farming practices. Previous studies have shown microbial colonization of the rumen epithelium in intensively fed goats from seven days to two years of age^11,12^. Spatial-temporal microbiota of compound stomachs in a pre-weaned goat model have also been described^13^. We suspected that the colonization of rumen microbiota would differ according to the diet fed to the animal. Until now, it is not clear how milk and roughage contribute to the overall composition and function of the rumen microbiome in grazing lambs, or how different microbes cooperate or compete with one another as the rumen environment changes due to changes in diet.

In this study, we performed 16 S rRNA amplicon sequencing on rumen fluid samples from 27 Tibetan lambs at nine ages (days (D) 0, 2, 7, 14, 28, 42, 56, 70, and 360, n = 3). Animals were divided into three groups according to their diet: preweaning (D0–14), milk and roughage (D14–70), and roughage only (D360). We described the structure and dynamics of the rumen microbial communities in the early stages of lamb development. We attempted to establish an evolutionary network of rumen bacteria with changes in diet and age. Our findings will strengthen the understanding of rumen microbiota function and provide insight into modulating ration formulations for early weaning of lambs in high-altitude grazing areas.

## Material and Methods

### Animal handling and sample collection

The ewes were raised at the Qinghai University experimental facilities. After delivery, lambs and ewes were housed separately during the day with access to the ewes for feeding three times per day. Lambs and ewes were kept together in the evening. The ewe’s milk was the sole feed available to lambs until D14. After D14, the lambs were grazed with the ewes, and pasture and breast milk were their only nutrition sources. The lambs did not received any formula granulated feed during the experiment. Fresh water was available for *ad libitum* consumption throughout the experimental period. Grazing lambs were naturally weaned at 4 months of age. Three lambs were slaughtered for each age time point (D0, D2, D7, D14, D28, D42, D56, D70, and D360, n = 3 for each group, total of 27 lambs). Rumen fluid was collected from lambs in 10 mL cryopreservation tubes. Rumens from D0 lambs were harvested and washed with a saline solution. Other ruminal samples were strained through four layers of cheesecloth. Ten milliliters of ruminal liquid were collected from each of the rest of the lambs immediately after birth and stored at −80 °C for further analysis.

### DNA extraction, PCR amplification and Illumina MiSeq sequencing

Genomic DNA samples were extracted using the CTAB method^14,15^. Purity and DNA concentration were detected by agarose gel electrophoresis. Samples were diluted with sterile water to 1 ng/μL in centrifuge tubes. Bacterial microbiota from the lamb rumen samples were investigated by sequencing the V4 hypervariable regions of the 16 S rDNA gene using the Illumina MiSeq platform. Sequencing was performed at the Novogene Bioinformatics Technology Co., Ltd. Briefly, DNA was amplified using the 515 F/806 R primer set (515 F: 5′-GTGCCAGCMGCCGCGGTAA-3′, 806 R: 5′-GGACTACHVGGGTWTCTAAT-3′). PCR was performed using Phusion high-fidelity PCR Master mix (New England Biolabs (Beijing) LTD., China) with the following conditions: 94 °C for 3 min (1 cycle), 94 °C for 45 s/50 °C for 60 s/72 °C for 90 s (35 cycles), and 72 °C for 10 min. PCR products were purified using the QIA quick Gel Extraction Kit (QIAGEN, Dusseldorf, Germany). The library was constructed using the Ion Plus Fragment Library Kit 48 rxns library (Thermo Fisher). The constructed library was subjected to Qubit quantification and library testing, and then sequenced using Ion S5TMXL (Thermo Fisher).

### Data analysis

Cutadapt V1.9.1^16^ was used to perform a low-quality partial cut on the reads. Sample data were separated from the reads according to their barcode. Reads were processed to detect and remove chimeric sequences using the UCHIME Algorithm^17,18^. The UPARSE method was used to cluster all clean reads^19^. By default, the sequences are clustered into operational taxonomic units (OTUs) with 97% identity. The sequence with the highest frequency in OTUs is selected as the representative sequence according to its algorithm principle. Specimen annotation of OTUs representative species annotation analysis with the Mothur method and SSUrRNA database of SILVA (http://www.arb-silva.de/)^20,21^ (set threshold of 0.8~1) allowed obtaining taxonomic information and count the populations of each sample at each classification level: kingdom, phylum, class, order, family, genus, species. composition. Fast multi-sequence alignment was performed using MUSCLE Version 3.8.31^22^ to obtain a systematic relationship of all representative OTUs. Finally, the data from each sample were homogenized using the least amount of data possible. Subsequent Alpha diversity analysis and Beta diversity analysis were conducted on the data after homogenization.

The Unifrac distance was calculated using QIIME Version 1.9.1, and a UPGMA tree was constructed. Principle coordinate analysis (PCoA) graphs were plotted using R Version 2.15.3^23^. PCoA analysis uses the RGC’s WGCNA, stats and ggplot2 packages. R software was used to analyze the difference between Beta diversity index groups. Parametric and non-parametric tests were performed. More than two groups, and the Wilcox test of the Agricolae package was used.

## Results

### Diversity index analysis of the rumen microbiome of growing lambs

**Figure 1:**
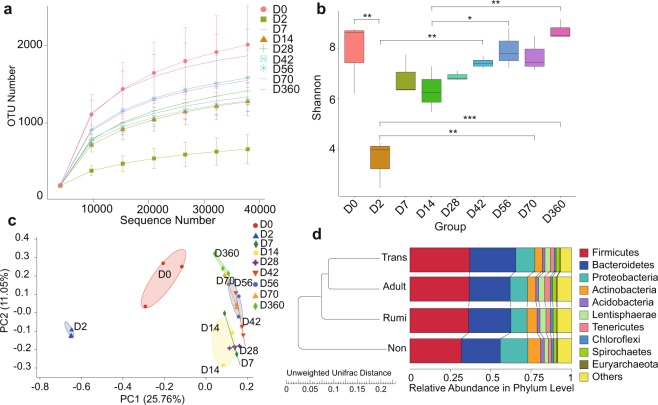
Diversity Index Analysis of the rumen microbiome. (**a**) Summary of rarefaction results based on operational taxonomic units (OTUs; 3% divergence) for each sample. (**b**) Comparison of diversity estimation (Shannon index) of the bacterial 16 S rRNA gene. (**c**) Principal coordinate analysis (PCoA) profile of microbial diversity across all samples using an unweighted UniFrac metric. The percentage of variation explained by PC1 and PC2 are indicated in the axis. (**d**) UPGMA (Unweighted Pair-group Method with Arithmetic Mean) analysis of the similarities between different samples. The UPGMA cluster tree based on the Unweighted Unifrac distance.

In total, we obtained 1,486,680 quality sequences from 27 samples (Table S1). These sequences included an average of 55,062 reads per sample. The total number of OTUs observed was 5,311 (Table S1). The rarefaction curves showed that the lambs’ rumen samples provided enough OTU coverage to accurately describe the bacterial composition of each group (Fig. 1a). This was also apparent with the Shannon diversity index, which indicated significant differences between groups (*P* < 0.05) (Fig. 1b).

Results of the PCoA analysis of the OTUs showed that the samples clustered according to age and demonstrated that rumen bacterial community colonization may be associated with age and diet. The average within-group similarity showed a significant difference between D0 and D2 (Fig. 1c). In addition, we divided the age components into four groups according to the stages of rumen development: non-ruminant stage (Non, D0, D2, D7, and D14); transition stage (Trans, D28, D42, and D56); ruminal stage (Rumi, D70) and adulthood (Adult, D360). At the phyla level, the non-ruminant group was significantly different from the other groups (Fig. 1d), With an abundance of *Proteobacteria* of 31.1%. The results showed that the early stage of rumen microbial colonization was dominated by *Proteobacteria*.

### Signature taxa at each age stage

**Figure 2:**
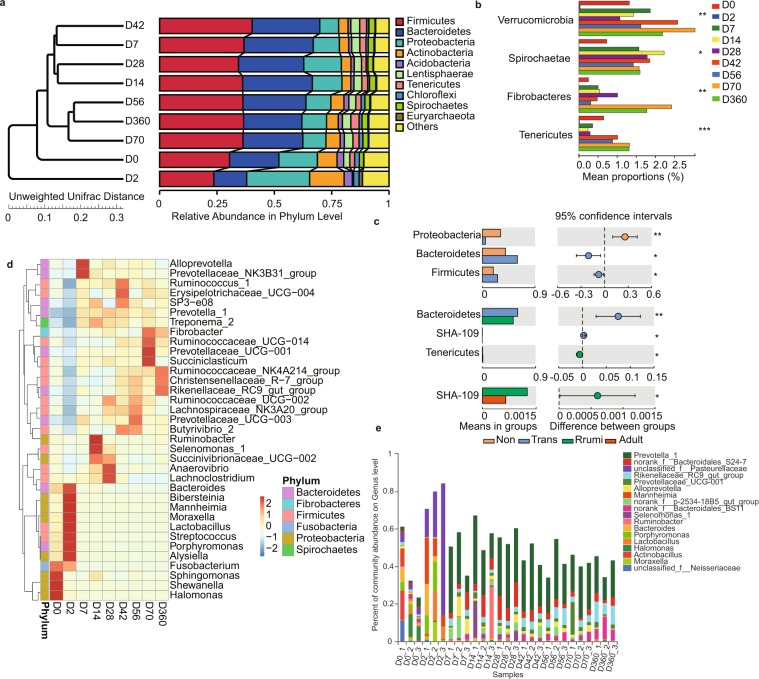
Distribution of bacterial composition in sheep rumens. (**a**) UPGMA (Unweighted Pair-group Method with Arithmetic Mean) analysis of the similarities between different samples at the phyla level. (**b**) Phylum level analysis of lamb rumen bacteria in different age groups. The ordinate indicates the species name under different classification levels, and the abscissa indicates the species abundance of the sample. Different colors represent different groups. (Kruskal-Wallis rank sum test; *0.01 < p ≤ 0.05, **0.001 < p < 0.01, ***p ≤ 0.001). (**c**) Wilcoxon rank-sum test bar plot on genus level of lamb’s rumen at different age stage (*0.01 < p ≤ 0.05, **0.001 < p < 0.01). (**d)** Community heatmap of the lamb rumen at genus level. The abscissa is the sample name, the ordinate is the species name, and the proportion of the species is represented by a certain color gradient. The right side of the Fig. is the value represented by the color gradient. (**e**) The community bar plot analysis of the lamb rumen at genus level of all samples (the abundance < 0.1 merged into others).

Next, we characterized the distinctive features of the rumen microbiome during the first year of life and defined signature taxa in different age groups. At the phyla level, thirty-nine phyla were identified within the rumen fluid samples (Supplementary Table 2). The top eleven most abundant phyla were *Proteobacteria*, *Bacteroidetes*, *Firmicutes*, *Actinobacteria*, *Fusobacteria*, *Spirochaetes*, *Lentisphaerae*, *Fibrobacteres*, *Chloroflexi*, *Acidobacteria* and *SHA-109* (Supplementary Table 2; Supplementary Fig. 5). We found that the four most abundant phyla in the rumen bacterial community made up more than 75% of the rumen bacterial community. *Bacteroidetes* and *Proteobacteria* were significantly changed during the first year of the growing lambs (Fig. 2a). *Bacteroidetes* abundance increased from 18.9% on D0 to 53.9% on D360 (*P* = 0.001) (Table S2). On the other hand, *Proteobacteria* abundance decreased significantly from 40.8% on D0 to 5.9% on D360 (*P* = 0.001) (Supplementary Table 2; Supplementary Fig. 5). Unweighted UniFrac distance analysis showed that ruminal microbial communities of 0 and 2-day old have a distinct cluster (Fig. 2a). In addition, *Verrucomicrobia*, *Spirochaetae*, *Fibrobacteres* and *Tenericutes* were relatively small proportions of the bacterial community and their abundance significantly increased with changes in age (*P* < 0.05). Interestingly, at 2 days of age, these bacteria are almost undetectable in the sample, and their abundance is close to 0 (Fig. 1b). This may be due to the intake of colostrum that rapidly changes the structure of the rumen bacterial community, but the microbial community gradually recovers over time. Secondly, at the family level, *Lactobacillaceae* abundance increased from 1.3% at D0 to 8.1% at D2, but rapidly decreased to 0.01% at D7 (Supplementary Fig. 1). The increased proportion of *Lactobacillaceae* likely occurred as a result of the disappearance of two days old related bacteria. To evaluate the differences in diets at different developmental stages, we compared the bacterial communities at the four developmental stages at the phyla level. Our results showed that the abundance of *Bacteroides* and *Firmicutes* were significantly increased, while the abundance of *Proteobacteria* was significantly decreased in the non-ruminant stage compared with the transition phase (Fig. 2c). *Bacteroidetes* and *SHA 109* abundance were significantly decreased, while *Tenericutes* was significantly increased in the transition stage compared with the ruminant stage (Fig. 2c). *SHA 109* was significantly decreased in the adult stage compared with the ruminant stage. Overall, we observed that different microbes appear at different physiological stages to achieve the functionalities of each specific period.

At the genus level, microbial communities underwent dramatic changes with the changes in age and diet. Dominant bacterial community heatmaps were drawn for each age (Fig. 2d). We found that specific genera of bacteria function at different ages, such as *Sphingomonas* (3.8%), *Shewanella* (1.6%) and *Halomonas* (5.7%) which were more abundant at 0 days of age. *Bacteroides* (6.9%), *Mannheimia* (13.7%), *Moraxella* (4.6%), *Lactobacillus* (8.1%), *Streptococcus* (4.0%) and *Porphyromonas* (8.6%) which were more abundant at 2 days of age (Supplementary Table 3); *Ruminococcaceae_NK4A214_ group* (1.9%), *Christensenellaceae_R–7_group* (2.8%) and *Rikenellaceae_RC9_ gut_ group* (9.0%) which were more abundant at 360 days of age. The relative abundance of all samples of different microbes at different developmental stages was plotted in the histogram reported in (Fig. 2e). This plot showed that *Prevotella_1* established an absolute advantage in the rumen after 7 days of age and Bibersteinia shows high abundance at 2 days of age but is not detected after 2 days of age. One-way ANOVA analysis showed the abundance of *Prevotella_1, norank_f__Bacteroidales_S24-7_ group, Rikenellaceae_RC9_ gut_ group, Prevotellaceae_UCG-003, Prevotellaceae_UCG-001, Bacteroidales_BS11_ gut_ group* and *Christensenellaceae_R-7_ group* increased significantly with developmental stage (*P* < 0.01) (Supplementary Fig. 2), these groups were long-term colonized in the rumen after 2 days old.

### Within-network interactions mirrored the bacterial microbiota relationships

**Figure 3:**
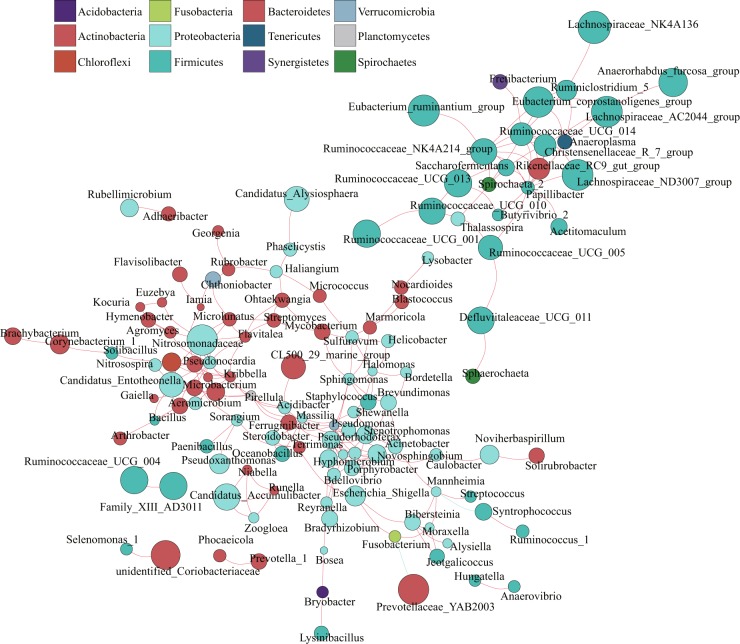
Network analysis applied to the lamb’s rumen microbiome. Genus correlation network maps mainly reflect the genus-relatedness of each taxonomic level under an environmental condition. The size of the node is proportional to the abundance of the genus. Node color corresponds to phylum taxonomic classification. Edge color represents positive (red) and negative (blue) correlations, and the edge thickness is equivalent to the correlation values.

To go deeper in the exploration of the interactions among rumen bacteria at different classification levels, a map of the rumen bacteria co-occurrence network was drawn. The co-occurrence network can be used to visualize the impact of different environmental factors on the adaptability of microbiomes. These dominant species and communities often play a unique and important role in maintaining microbial community structure and stability in the environment^24^. According to the results of the analysis, there was a positive correlation between *Bacteroidetes* and *Proteobacteria*, and between *Fusobacteria* (*Fusobacterium*) and *Proteobacteria*. *Fusobacteria* (*Fusobacterium*) was negatively correlated with *Bacteroidetes* (*Prevotellaceae_YAB2003*). *Firmicutes* (*Syntrophococcus*) was negatively correlated with *Proteobacteria* (*Mannheimia*) (Fig. 3). In addition, as suspected, the highly abundant bacteria in the rumen such as *Lachnospiraceae_NK4A136, Ruminococcaceae_UCG_013, Christensenellaceae_R-7_ group, Rikenellaceae_RC9_gut_ group, Eubacterium_ruminantium_ group, Prevotellaceae_UCG-003* and *Prevotellaceae_UCG-001* were all positively correlated, but Prev*otella_1* only had a positive correlation with *Phocaeicola* (Fig. 3). As shown by the co-occurrence network, the different components of the rumen present a complex synergistic and cumbersome relationship. The microscopic world of the rumen is more complex than demonstrated in this study. It may also include a collaborative network of bacteria-fungi-protozoa-virus-phage, which requires more in-depth research.

**Figure 4:**
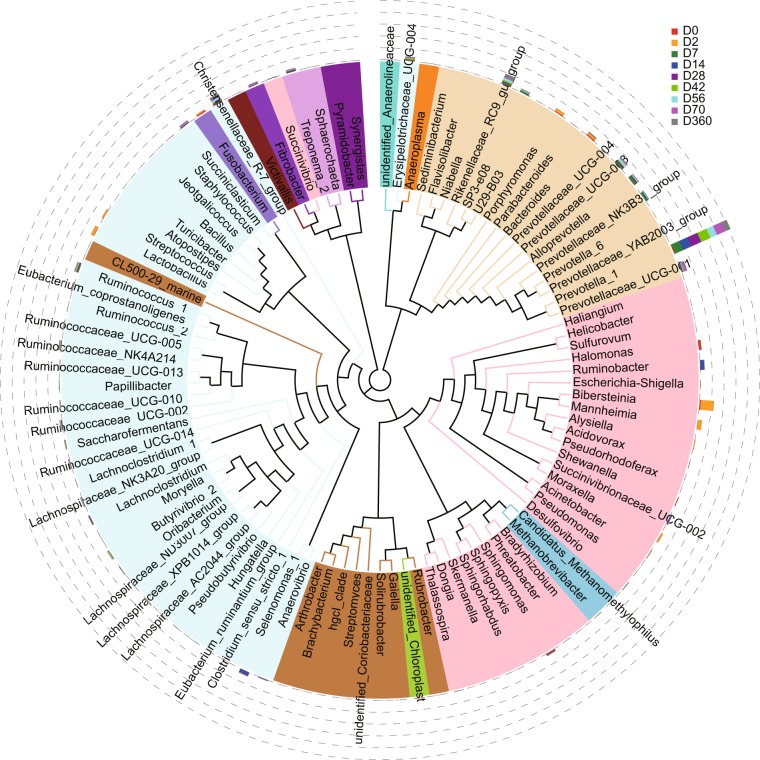
Phylogenetic tree on genus level. Each branch in the phylogenetic tree represents a species, and the length of the branch is the evolutionary distance between the two species. The histogram outside the circle shows the relative proportion of reads belonging to different species in each group. Different colors of circles represent different phyla.

Next, to further understand the relationships among rumen bacterial at different developmental stages, a phylogenetic tree was constructed using representative sequences of bacterial genera (Fig. 4). We found that bacteria with high genetic similarity play a mutually reinforcing role in the colonization at each stage. For example, *Bibersteinia*, *Mannheimia*, and *Shewanella* have close kinship. Coincidentally, they have higher abundance at D2 (Fig. 4). *Prevotellaceae_UCG-004*, *Prevotellaceae_UCG-003*, *Prevotella_1* and *Alloprevotell*a have close kinship and exhibited higher abundance at D7 (Fig. 4). There may be a phenomenon in which bacteria with close kinship are preferentially colonized, but stronger evidences are needed to justify this phenomenon.

## Discussion

This study characterized the dynamics and stabilization of the sheep rumen microbiome during the first year of life. Both the maturity of the rumen and the development of rumen microbiota is critical for ruminant livestock production. Our results showed significant differences in bacterial richness and diversity during the first year of life and demonstrated bacterial dynamics of the colonization process at several major developmental stages. During the first year of growth, lambs undergo several major dietary changes from colostrum intake to crude fiber intake to a diet independent of their mother’s milk postweaning. These stages have a great impact on rumen microbiota colonization. Although colonization of the microbiota has a significant relationship with the host^25^, environment^26^, diet^27^, age^13^, and other factors, the impact of diet on the microbiota community is also very large^8^.

**Figure 5:**
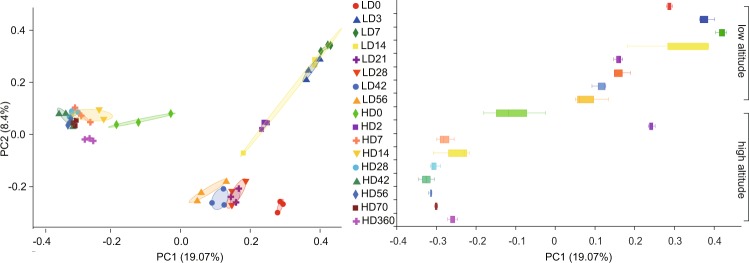
Beta diversity assessment of differences in lamb rumen microbial communities under different feeding conditions. “H” stands for high-altitude sheep and “L” stands for low-altitude goats. Points of different colors or shapes represent samples of different groups. The closer the two sample points, the more similar the composition of the sample species. The box plots in the Fig. represent the dispersion of the distribution of different sets of samples on the PC1 axis.

Our results also confirmed that both diet and age have great effects on rumen microbial community composition. Prior to this, other studies have investigated the colonization process of the bovine rumen microbiome from birth to adulthood, and reported a convergence toward a mature bacterial arrangement with age^28^. A previous study has also investigated the rumen microbiota of pre-ruminant calves using metagenomic tools, which revealed that their rumen microbiota displayed considerable compositional heterogeneity during early development. Rumen microbiome studies on goats, indicated the relative abundance of Firmicutes was stable from D7 to D720. On the contrary, our study found that before D7, the abundance of *Firmicutes* changed significantly, but stabilized after D7. Secondly, the microbial species and abundance of the rumen contents are different from those of the rumen epithelium. Accordingly, the previous study found bacterial diversity of the rumen epithelium during development and that colonization of the rumen epithelium is related to age and may contribute to anatomic and functional development of the rumen^12^. To characterize the contribution of age and diet to rumen microbial age-related succession, we used gene sequencing data from pre-weaned goats (D0–D56) to compare with our data and found that living environment and genotype shaped different types of microbial colonization at the same age (Fig. 5) (Zhang *et al*. 2018). At the early stage, living environment and genotype represent the main influencing factors for the difference in rumen microbial colonization.

A previous study found that diet influencing the structure and function of the neonatal gut microbial, is an important driver of microbial colonization in kids^29^. The study of rumen microbial age-related succession at an early phase of life is influenced by changes in both age and diet. Indeed, it has been shown that the key turning point in gastrointestinal microbial colonization is the introduction of solid food in the diet^30,31^, With the consequence that the established ruminal bacterial community resulted shaped by the solid food arrival^30^. We thus focused our investigations on the microbiome-host crosstalk in early life. Ruminal microbiome-host crosstalk stimulates the development of the ruminal epithelium in neonatal lambs, and the early introduction of feed significantly promotes rumen epithelium development. It has been also demonstrated that early diet intervention is essential for rumen development^32^. To familiarize the most sensitive time window of dietary innervations at early stages, it is necessary to record the colonization progress of rumen microbes of lambs under natural conditions (grazing). In this study doesn’t have any dietary intervention. Here, we showed that the rumen bacterial community of grazing sheep can maintain stability from birth to 3–4 weeks, indicating that this time is crucial for dietary intervention before birth to 3–4 weeks. However, the long-term influence of early life nutritional intervention in relation to rumen development is still largely unknown and some aspects need to be carefully considered, including the composition of the starter food, the type of forage and the timing of its introduction.

In addition, microbes were present in the rumen of lambs that did not consume colostrum after birth. The most abundant genera were *Actinobacillus* (6.1%), *Halomonas* (5.7%), *Mannheimia* (3.8%), *Sphingomonas* (3.8%), and *Lactobacillus* (1.3%). It is possible that the source of these bacteria may be the mother’s vagina^33^, skin^34^, and the environment. The role of *Lactobacillus* is to lower the pH in the gastrointestinal tract and prevent the colonization of harmful bacteria^35^. When colostrum is ingested, ruminal microbes have undergone major changes. Previous studies found the following bacterial populations present in goat colostrum samples, *Lactococcus lactis*, *Lactobacillus delbrueckii*, *Lactobacillus fermentum, Pediococcus acidilactici*, and others belonging to the *Staphylococcus, Enterobacter* and *Escherichia genera*^36,37^. In our sequencing results, we found that the *Lactococcus* abundance is relatively high in D2. Rumen fluid pH was lower than 4.0, indicating that colostrum intake reduced rumen pH and inhibited the growth of harmful bacteria, but that rumen fluid pH gradually recovered.

Since grazing alongside the ewes after 14 days of age, the lambs gradually begin to consume crude fiber via their forage intake, stimulating major changes in the rumen microbes. The primary microbes affected were the bacteria of the digestive group. *Prevotella_1*, *Bacteroidales_S24_7*, *Ruminobacter* and *Selenomonas* (>5%) had greater abundance after D14. A previous study found that the association of *Prevotella* with a plant-rich diet suggested that *Prevotella* is a beneficial genus of microbes^38^. Members of the *S24-7* family are also differentiated by their degree of IgA-labeling suggesting at least some members of the group are targeted by the innate immune system^39^. While these observations are currently limited to murine studies, they suggest that *S24-7* is involved in host-microbe interactions that impact gut function and health^40^. Members of *Selenomonas* affect L-lactate utilization^41^, with the intake of crude fiber, rumen fermentation produces a large amount of lactic acid, which requires the corresponding bacterial regulation and absorption. The genus *Bacteroides* is the most abundant genus in newborn ruminant, while *Prevotella* is the main genus observed after D7 ruminant^28^. This microbial succession has been associated with dietary changes, from a primarily milk-based diet to a forage-based diet^28,42^. it is clear that changes in diet and feeding behavior can also lead to changes in the rumen shape and functionality, eventually leading to a rumen microbiome. Interestingly, in nursing ruminants, a special structure called the reticular groove directs milk t into the abomasum bypassing the rumen. Cellulose bacteria *Ruminococcus flavefaciens* were detected in the rumen of 1-day-old and 3-day-old ruminant^6,28^. To clarify this question, further efforts are needed for understanding relationships between microbial composition and changes to biological activity to host phenotype and host physiology. Further efforts are also needed to better understand how the rumen microbiome of young ruminants’ changes over time, with large biological replicated and strict diet control. In this study, each group consisted of 3 replicates, the minimum number to analyze the differences between individual samples within the group. We showed gene similarities and differences in bacterial community composition in the heatmap showed in (Supplementary Fig. 3). We also provided the ANOSIM analysis to test whether the difference between groups is significantly greater than the difference within the groups (ANOSIM, R = 0.61, *P* = 0.001; Supplementary Fig. 4). We found a small variation within the groups. Despite a lot of “omics” works on the rumen have been performed, longitudinal studies are needed to examine how the rumen microbiome changes over time, and how it can affect host productivity.

In this study, we performed 16 s rRNA gene sequencing on lamb rumen fluid to identify rumen microbiome signature characteristics during the first year of life. We found that rumen microbe populations stabilize after 28 days of age and dynamic changes in bacterial colonization occur as a result of changes in diet before 28 days of age. Our results also underscore the role of colostrum in the shaping and succession of the rumen microbial community during the first year of life, and the ability of *Lactobacillus* to significantly regulate rumen microbial structure. In addition, we found that the dietary environment is more important for the colonization of rumen microbial community with change of age. This study provides new insights into the colonization of bacterial communities in lambs that may be useful in designing strategies to promote colonization of target communities to improve functional development.

### Ethics approval and consent to participate

All procedures for animal experiment were conducted according to the guidelines approved by the Animal Care Committee (Approval Number: NQH14023), Institutional Animal Care and Use Committee of the Qinghai Academy of Animal Science and Veterinary Medicine. The principles of laboratory animal care were met, and slaughter procedures were performed in accordance with the guidelines of Chinese national standards of cattle and goat slaughtering by reducing the animal suffering as much as possible. All experimental protocols were also approved by Institutional Animal Care and Use Committee of the Qinghai Academy of Animal Science and Veterinary Medicine.

## Supplementary information

Supplementary Information

Supplement Table 1

Supplement Table 3

## Data Availability

Amplicon sequences generated were entered into the National Center for Biotechnology Information (NCBI) under accession numbers SRA: SRP162909.
