# Supplementary material for: Dynamics and stabilization of the rumen microbiome in yearling Tibetan sheep
Source: Sci Rep. 2019 Dec 23;9:19620. doi: 10.1038/s41598-019-56206-3 (PMC6927978; doi:10.1038/s41598-019-56206-3)
Supplement: Supplementary file 1 — Supplementary Information [file 41598_2019_56206_MOESM1_ESM.docx]

**Title: Dynamics and stabilization of the** **rumen microbiome** **in yearling Tibetan sheep**

Lei Wang†^1,2^, Ke Zhang†^3^, Chenguang Zhang^3^, Yuzhe Feng^1^, Xiaowei Zhang^1^, Xiaolong Wang^3^, Guofang Wu^1,2^*

^1^Academy of Animal Science and Veterinary Medicine, Qinghai University, Xining, 810016, China.

^2^State Key Laboratory of Plateau Ecology and Agriculture, Qinghai University, Xining, 810016, China.

^3^College of Animal Science and Technology, Northwest A&F University, Yangling, 712100, China.

†These authors contributed equally to this work.

*Correspondence: Guofang Wu (G.W)

Email: [letitbe521@163.com](mailto:letitbe521@163.com).

Tel/Fax: +86-0971-6254161.

**Supplementary Table 1 Statistics for the sequencing data of 27 lamb rumen fluid samples.**

See excel of supplement Table 1.

**Supplementary Table 2 Phylum level analysis of lamb's rumen bacteria in different age groups.**

| Tax | D0 (%) | D2 (%) | D7 (%) | D14 (%) | D28 (%) | D42 (%) | D56 (%) | D70 (%) | D360 (%) | *P* | Corrected *P*-value |
| --- | --- | --- | --- | --- | --- | --- | --- | --- | --- | --- | --- |
| p__Bacteroidetes | 18.850 | 18.880 | 64.140 | 52.630 | 62.200 | 58.830 | 57.920 | 52.080 | 53.880 | 0.012 | 0.131 |
| p__Firmicutes | 21.150 | 14.060 | 23.140 | 22.790 | 22.750 | 29.930 | 28.210 | 29.480 | 27.840 | 0.212 | 0.432 |
| p__Proteobacteria | 40.790 | 59.770 | 4.656 | 18.300 | 8.199 | 2.785 | 4.527 | 3.749 | 5.940 | 0.031 | 0.185 |
| p__Actinobacteria | 5.931 | 1.127 | 1.388 | 0.515 | 0.576 | 0.295 | 1.729 | 2.057 | 1.674 | 0.703 | 0.721 |
| p__Verrucomicrobia | 1.318 | 0.035 | 1.865 | 1.427 | 1.073 | 2.578 | 1.618 | 3.031 | 2.187 | 0.059 | 0.209 |
| p__Spirochaetae | 0.729 | 0.002 | 1.566 | 2.226 | 1.785 | 1.853 | 1.423 | 1.573 | 1.598 | 0.270 | 0.502 |
| p__Chloroflexi | 0.946 | 0.102 | 0.913 | 0.367 | 1.126 | 1.326 | 1.456 | 1.761 | 0.459 | 0.503 | 0.599 |
| p__Fibrobacteres | 0.255 | 0.001 | 0.507 | 0.546 | 1.011 | 0.481 | 0.315 | 2.420 | 1.774 | 0.033 | 0.185 |
| p__Fusobacteria | 3.356 | 2.682 | 0.048 | 0.054 | 0.048 | 0.041 | 0.044 | 0.050 | 0.072 | 0.045 | 0.194 |
| p__Tenericutes | 0.652 | 0.013 | 0.363 | 0.240 | 0.294 | 1.014 | 0.881 | 1.320 | 1.313 | 0.010 | 0.131 |
| p__unclassified_k__norank | 1.087 | 2.792 | 0.482 | 0.245 | 0.095 | 0.453 | 0.244 | 0.251 | 0.304 | 0.684 | 0.721 |
| p__Acidobacteria | 1.586 | 0.105 | 0.130 | 0.070 | 0.153 | 0.066 | 0.692 | 0.651 | 0.558 | 0.732 | 0.732 |
| p__Gemmatimonadetes | 0.969 | 0.092 | 0.038 | 0.034 | 0.031 | 0.026 | 0.384 | 0.429 | 0.473 | 0.359 | 0.543 |
| p__Cyanobacteria | 1.105 | 0.065 | 0.166 | 0.064 | 0.066 | 0.065 | 0.099 | 0.446 | 0.117 | 0.362 | 0.543 |
| p__Synergistetes | 0.171 | 0.000 | 0.326 | 0.350 | 0.224 | 0.111 | 0.123 | 0.184 | 0.143 | 0.084 | 0.234 |
| p__Planctomycetes | 0.227 | 0.016 | 0.106 | 0.030 | 0.122 | 0.025 | 0.084 | 0.138 | 0.771 | 0.560 | 0.643 |
| p__Lentisphaerae | 0.074 | 0.001 | 0.054 | 0.041 | 0.078 | 0.074 | 0.063 | 0.084 | 0.455 | 0.042 | 0.194 |
| p__Nitrospirae | 0.256 | 0.018 | 0.018 | 0.010 | 0.050 | 0.010 | 0.053 | 0.103 | 0.090 | 0.345 | 0.543 |
| p__Tectomicrobia | 0.188 | 0.018 | 0.000 | 0.001 | 0.002 | 0.001 | 0.047 | 0.060 | 0.062 | 0.415 | 0.580 |
| p__Armatimonadetes | 0.095 | 0.011 | 0.029 | 0.004 | 0.002 | 0.006 | 0.047 | 0.045 | 0.056 | 0.309 | 0.523 |
| p__Chlorobi | 0.095 | 0.010 | 0.040 | 0.001 | 0.001 | 0.000 | 0.004 | 0.008 | 0.010 | 0.056 | 0.209 |
| p__Elusimicrobia | 0.023 | 0.002 | 0.002 | 0.014 | 0.013 | 0.008 | 0.004 | 0.044 | 0.043 | 0.071 | 0.232 |
| p__Saccharibacteria | 0.018 | 0.009 | 0.004 | 0.002 | 0.007 | 0.012 | 0.014 | 0.008 | 0.070 | 0.505 | 0.599 |
| p__WA-aaa01f12 | 0.013 | 0.000 | 0.003 | 0.008 | 0.011 | 0.008 | 0.006 | 0.011 | 0.069 | 0.026 | 0.185 |
| p__unclassified | 0.055 | 0.065 | 0.000 | 0.003 | 0.001 | 0.000 | 0.001 | 0.000 | 0.001 | 0.079 | 0.234 |
| p__Gracilibacteria | 0.008 | 0.109 | 0.000 | 0.000 | 0.000 | 0.000 | 0.000 | 0.000 | 0.000 | 0.005 | 0.131 |
| p__Chlamydiae | 0.004 | 0.001 | 0.006 | 0.008 | 0.042 | 0.000 | 0.000 | 0.000 | 0.000 | 0.118 | 0.304 |
| p__Deinococcus-Thermus | 0.008 | 0.003 | 0.005 | 0.009 | 0.009 | 0.001 | 0.004 | 0.001 | 0.003 | 0.143 | 0.328 |
| p__SR1__Absconditabact | 0.001 | 0.000 | 0.000 | 0.000 | 0.000 | 0.000 | 0.001 | 0.000 | 0.035 | 0.013 | 0.131 |
| p__Latescibacteria | 0.013 | 0.000 | 0.004 | 0.002 | 0.000 | 0.001 | 0.002 | 0.005 | 0.005 | 0.215 | 0.432 |
| p__TM6__Dependentiae_ | 0.006 | 0.001 | 0.000 | 0.002 | 0.012 | 0.000 | 0.001 | 0.001 | 0.003 | 0.446 | 0.580 |
| p__Omnitrophica | 0.006 | 0.001 | 0.001 | 0.001 | 0.010 | 0.000 | 0.000 | 0.000 | 0.001 | 0.307 | 0.523 |
| p__Ignavibacteriae | 0.003 | 0.001 | 0.000 | 0.000 | 0.011 | 0.000 | 0.000 | 0.000 | 0.000 | 0.222 | 0.432 |
| p__BRC1 | 0.008 | 0.000 | 0.001 | 0.001 | 0.000 | 0.001 | 0.001 | 0.001 | 0.002 | 0.125 | 0.304 |
| p__GAL15 | 0.002 | 0.002 | 0.000 | 0.000 | 0.000 | 0.000 | 0.002 | 0.003 | 0.000 | 0.683 | 0.721 |
| p__FBP | 0.001 | 0.000 | 0.000 | 0.000 | 0.000 | 0.000 | 0.004 | 0.002 | 0.001 | 0.683 | 0.721 |
| p__Hydrogenedentes | 0.000 | 0.000 | 0.001 | 0.002 | 0.000 | 0.000 | 0.000 | 0.000 | 0.000 | 0.507 | 0.599 |
| p__Parcubacteria | 0.000 | 0.000 | 0.000 | 0.000 | 0.003 | 0.000 | 0.000 | 0.000 | 0.000 | 0.434 | 0.580 |
| p__SBR1093 | 0.002 | 0.000 | 0.000 | 0.000 | 0.000 | 0.000 | 0.000 | 0.000 | 0.000 | 0.434 | 0.580 |

**Supplementary Table 3: Genus level analysis of lamb rumen bacteria across age groups.**

See excel of supplement Table 3.


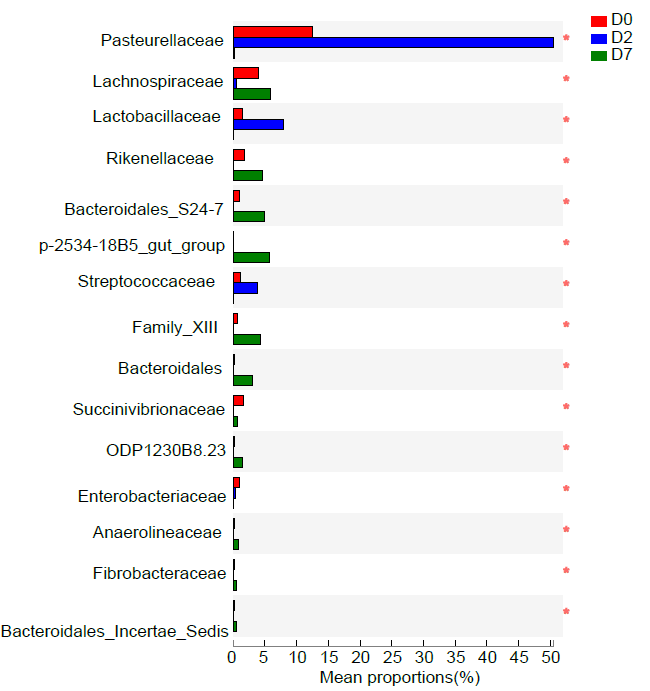
**Supplementary Figure 1.** Family level analysis of lamb rumen bacteria on D0, D2 and D7. The ordinate indicates the species name under different classification levels, and the abscissa indicates the sample species abundance. Different colors represent different groups. (Kruskal-Wallis rank sum test; * 0.01<p≤0.05, ** 0.001< p <0.01).

**
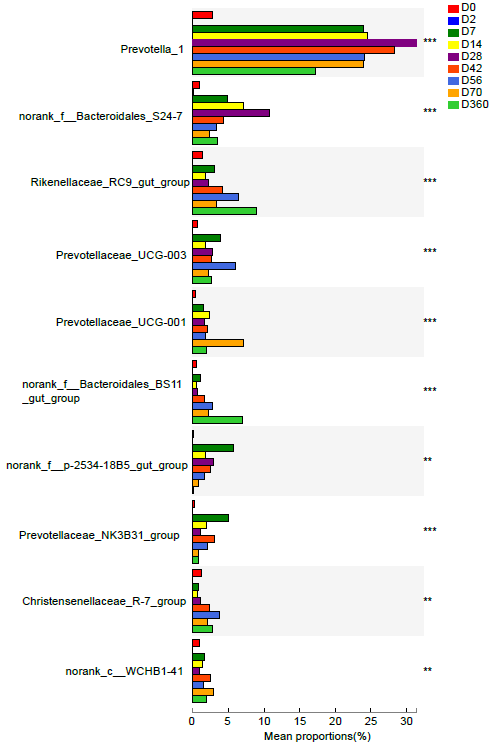
Supplementary Figure** **2.** Test of significant differences between genera in lamb rumen bacteria. The ordinate indicates the species name under different classification levels, and the abscissa indicates the abundance of a species. Different colors represent different groups. (* 0.01 < p ≤ 0.05, ** 0.001 < p < 0.01, *** p ≤ 0.001).

**Supplementary Figure 3.** Heatmap representation of the top 50 genera under different age group. The relative values are represented by color intensity according to the legend provided right the

figure.
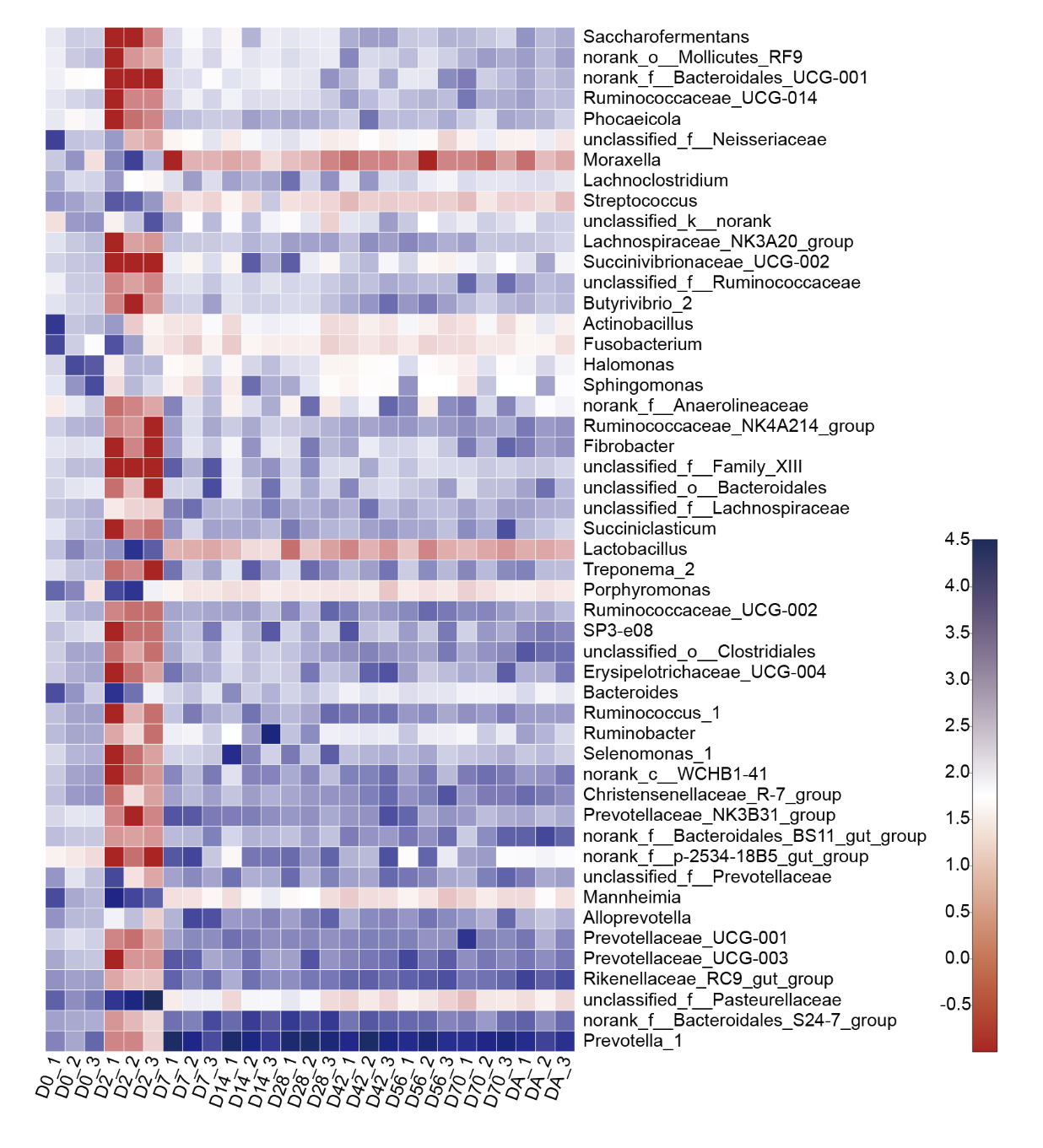


**Supplementary Figure 4.** The ANOSIM analysis of different group.


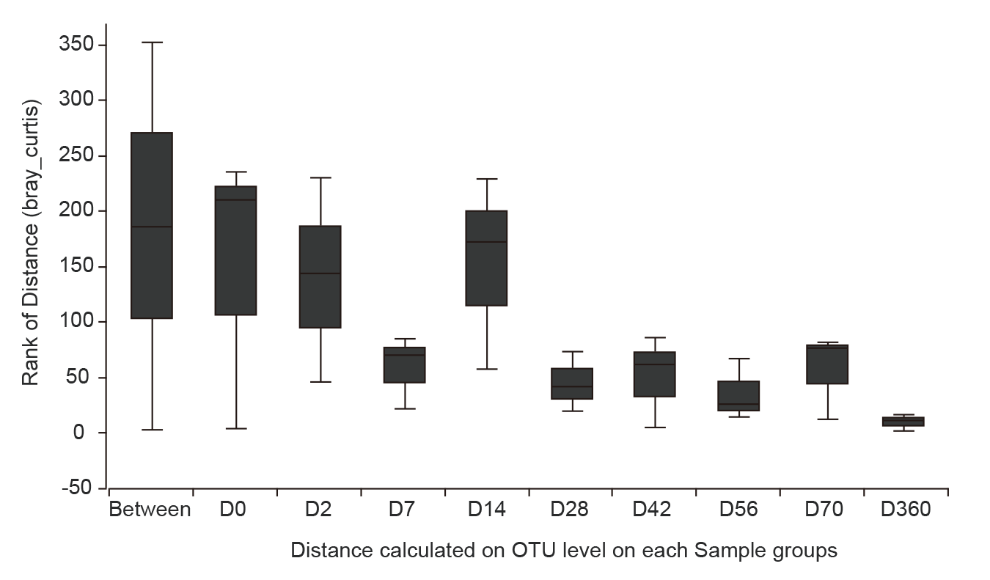


**Supplementary Figure 5. Percent of community abundance on phylum level of all samples.**

**
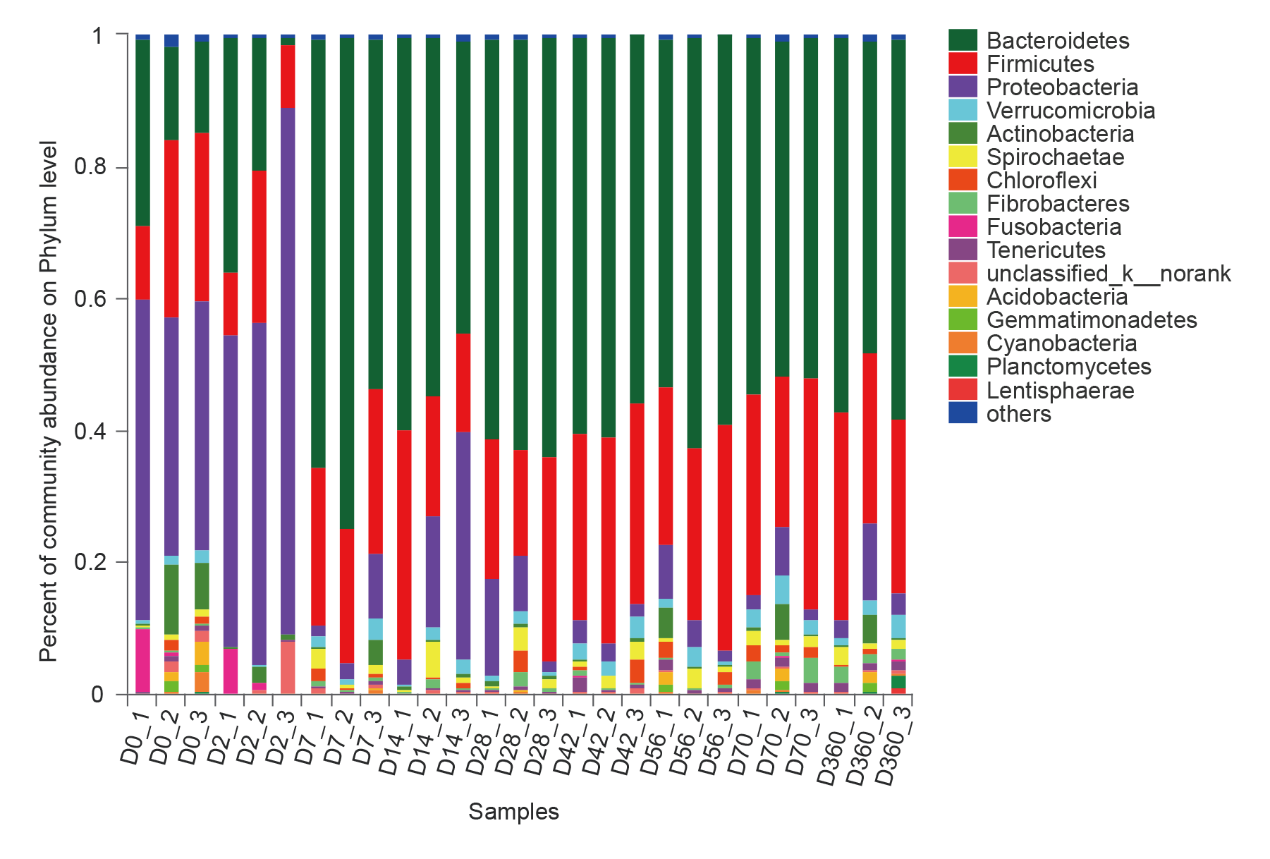
**
